# Bile acid metabolism and signaling: potential therapeutic target for nonalcoholic fatty liver disease

**DOI:** 10.1038/s41424-018-0034-3

**Published:** 2018-06-29

**Authors:** Chuan-Hao Lin, Rohit Kohli

**Affiliations:** Division of Gastroenterology, Hepatology and Nutrition, Children’s Hospital Los Angeles, and the Department of Pediatrics Keck School of Medicine, University of Southern California, Los Angeles, CA USA

## Introduction

Nonalcoholic fatty liver disease (NAFLD) has emerged as a leading cause of chronic liver disease^1^. We now understand that NAFLD affects one out of every four people in the world (global prevalence 25.24%), thus in fact making it the most prevalent of all liver diseases and as such, a major public health problem^2, 3^. The disease is defined by abnormally increased fat (>5% steatosis) in hepatocytes, which can manifest in some with additional features of inflammation and cell damage or nonalcoholic steatohepatitis (NASH). NASH can present with progressive stages of fibrosis and even cirrhosis. NAFLD is generally asymptomatic and only 10–15% have findings on biopsy consistent with NASH, and an even a smaller number, 5% of individuals develop cirrhosis, with 1–2% requiring liver transplant^2^. However, because of the enormous denominator of individuals with NAFLD, this 1–2% is estimated to have resulted in nearly 20 million liver-related deaths to date^2^. These data clearly underscore the need of effective treatment especially for the 5% of NAFLD with NASH.

## Approach to treating NAFLD and NASH

Multiple drugs are currently in Phase II/III clinical trials for NASH. Although disparate mechanisms are being explored, the therapeutic agents may be grouped into four broad categories: (1) bile acid metabolism/farnesoid X receptor (FXR) signaling pathway, (2) anti-diabetic/lipid, (3) anti-apoptotic agents, and (4) anti-inflammatory/anti-fibrotic agents. There has been recent work investigating the therapeutic potential of bile acids and bile acid receptor agonists to treat NAFLD^4, 5^. Herein we review these key basic science articles to discuss the potential use of those agents in treating NAFLD.

## Bile acids signaling and mechanism for NASH

Bile acids are steroid molecules synthesized in the liver from cholesterol and excreted into bile. Traditionally, the functions of bile acids are thought to stimulate hepatic bile flow and to aid digestion and absorption of fats from the intestinal lumen^6^. However, recent studies have shown that bile acids may function as signaling molecules through a variety of receptors to regulate their own synthesis as well as other metabolic processes, such as glucose, lipids, and energy homeostasis^7^. The regulatory functions of bile acids are mediated through specific bile acid-activated receptors, including members of the nuclear receptor superfamily (farnesoid X receptor [FXR; NR1H4], vitamin D receptor [VDR; NR1I1], pregnane X receptor [PXR; NR1I2]), and members of the G-protein-coupled receptor superfamily (Takeda G-protein-coupled receptor 5 [TGR5] and sphingosine-1-phosphate receptor 2)^8^. FXR, named for its ability to bind farnesoid, is a nuclear bile acid receptor. Bile acids are identified as natural ligands for FXR and bile acids activate FXR in a manner predicted for nuclear receptor ligands^9^. Bile acids and other agents that activate FXR are attractive because they affect many pathways that may be involved in the NASH pathogenesis. Bile acids have broad and powerful hormonal properties as gene regulators that parallel their physiologic roles in choleresis and digestion^10, 11^. It has been shown in both cell and animal studies that bile acids modulate insulin signaling and can improve insulin resistance, mediated in part by activation of FXR by bile acids^12^. Bariatric surgical procedures, such as vertical sleeve gastrectomy (VSG) are currently accepted to be the most effective and durable therapy for obesity^13^. VSG is also associated with reversal and improvements in obesity-related co-morbidities, including type-2 diabetes (T2DM) and NASH. To identify the molecular mechanisms responsible for weight loss and other metabolic improvements, Ryan et al.^4^ examined the results of VSG surgery applied to mice with diet-induced obesity and targeted genetic disruption of FXR. The results showed that VSG is associated with increased circulating total serum bile acids and changes to gut microbiota. In the absence of FXR signaling (Fxr-knockout mice subject to VSG), the ability of VSG to reduce food intake, effectively produces body weight loss and improved glucose tolerance was substantially reduced. Ryan et al. reported that the improvement in T2DM end points correlated with increases in Roseburia species of microbiota, while in other papers from the same group, weight lost was linked to the level of serum bile acid elevation post VSG^14^, while NASH improvement post VSG was dependent on an intact FXR–SHP (small heterodimer partner) signaling pathway^15^. Together, these papers point to the importance of the bile acid-signaled FXR pathway in the metabolic improvement observed after VSG.

## Human studies of FXR agonists for the treatment of NAFLD

To date, there have been two human trials of FXR agonists for the treatment of NAFLD/NASH. Obeticholic acid (OCA) is a derivative of chenodeoxycholic acid and is a highly potent FXR agonist. One of these trials was a proof of concept phase 2a, double-blind, placebo-controlled study of two doses of OCA in patients with NASH and T2DM^16^. The results showed OCA increased insulin sensitivity, and reduced markers of liver inflammation and fibrosis. However, the study also showed an increase in low-density lipoprotein cholesterol (LDL) and a concomitant reduction in circulating high-density lipoprotein cholesterol (HDL), a lack of change in plasma alanine amino transferase and enhanced liver fibrosis scores was notable.

The other trial is a phase 2b clinical trial based on histological endpoints that was performed by the National Institutes of Health supported NASH Clinical Research Network. Neuschwander-Tetri et al. reported on the effects of OCA in patients with NASH, in a placebo-controlled, randomized trial (FLINT)^17^. Results showed OCA improved the histological features of NASH, but its long-term benefits and safety needed further clarification. Patients were treated for 72 weeks of OCA, and the primary endpoint was an improvement in histology, as measured by a two-point reduction in a composite activity histological score without worsening of fibrosis. The planned interim analysis for safety and efficacy showed that OCA had significant beneficial effects on NASH-related liver health. However, this interim analysis also found unanticipated increases in LDL and decreased HDL. Because of these factors, the termination of therapeutic phase of the trial was recommended by the study’s Data Safety and Monitoring Board (DSMB) and concurred upon by the study sponsor and NIH (NIH News Releases, November 7, 2014, https://www.nih.gov/news-events/news-releases/new-drug-common-liver-disease-improves-liver-health). In addition to OCA, other FXR agonists that are being investigated include GS-9674 (Phase II a randomized, clinical trial in patient with NAFLD, ClinicalTrial.gov identifier: NCT01999101).

## Inhibition and importance of normal bile acid enterohepatic circulation

Bile acids are synthesized from cholesterol in the liver, excreted into bile to facilitate lipid absorption in the intestine. About 95% of intestinal bile acids are reabsorbed in the ileum by the apical sodium-dependent bile acid transporter (ASBT) and conveyed through the portal vein to the liver, where they are taken up by hepatocytes to be re-secreted into bile. There is a well-described post-prandial rise in serum bile acids^18^ and also the bile acid-stimulated ileal FXR triggers the production and secretion of enteric endocrine hormone, fibroblast growth factor (FGF) 19, or its rodent ortholog FGF 15^19^. Bile acids signaling in the intestine and liver have a role in the regulation of lipid, glucose, and energy homeostasis, and is a potential target for the treatment of obesity and NAFLD^20^. Using the animal model with high-fat diet (HFD)-fed mice, Rao et al. interrupted the enterohepatic bile acid circulation using a luminally restricted ASBT inhibitor (ASBTi; CS-435)^5^. The administration of this ASBTi increased fecal bile acid excretion and mRNA expression of bile acid synthesis genes in the liver and reduced mRNA expression of ileal bile acid-responsive genes including Fgf 15. Further, ASBT inhibition restored glucose tolerance, reduced hepatic triglyceride and total cholesterol concentrations, and improved NAFLD activity score in HFD-fed mice. The study suggests that blocking ASBT function with a luminally restricted inhibitor can improve both hepatic and whole body aspects of NAFLD. These murine data are, however, contrary to previous human work, wherein use of a luminal bile acid-binding resin, colesevelam (a bile acid sequestrant), was reported to produce increased hepatic steatosis by magnetic resonance evaluation^21^. Further, multiple authors have shown that obese individuals have lower circulating serum bile acid levels and an impaired post-prandial bile acid and FGF19 response^22^. The circulating bile acid level and post-prandial responses of FGF19 and bile acids are both corrected with  surgically induced weight loss (e.g. VSG). Clinical trials with inhibitor of ASBT (SHP626-Volixibat, ClinicalTrial.gov identifier: NCT02787304) are ongoing. Clearly, more work is needed to clarify the role of bile-acid recycling and metabolism in obesity.

## Conclusion

Bile acids and other agents that activate FXR and other related pathways may be involved in NASH pathogenesis. Studies into the mechanisms of bariatric surgery (VSG) clearly showed the importance of the bile acid-signaled FXR pathway in resultant metabolic improvement^4^. FXR agonists and ASBT inhibitors are in various phases of testing though given the preliminary issues identified with lipid metabolism and hepatic steatosis beg the question for further more targeted work.
